# Ion Exchange Dynamics in Cerium Nitrate Solution Regulated by Remotely Activated Industrial Ion Exchangers

**DOI:** 10.3390/ma14133491

**Published:** 2021-06-23

**Authors:** Talkybek Jumadilov, Laila Yskak, Aldan Imangazy, Oleg Suberlyak

**Affiliations:** 1 Laboratory of Synthesis and Physicochemistry of Polymers, Bekturov Institute of Chemical Sciences, 106 Valikhanov Str., Almaty 050010, Kazakhstan; Jumadilov@mail.ru (T.J.); leila_23.93@mail.ru (L.Y.); 2 Department of Chemistry, Kazakh National Women’s Teacher Training University, 99 Ayteke bi Str., Almaty 050000, Kazakhstan; 3 School of Chemical Engineering, Kazakh-British Technical University, 59 Tole bi Str., Almaty 050000, Kazakhstan; 4 Department of Chemical Technology of Plastics Processing, Lviv Polytechnic National University, 12 Bandera Str., 79013 Lviv, Ukraine; suberlak@polynet.lviv.ua

**Keywords:** ion exchangers, interpolymer system, remote interaction, cerium ions, sorption

## Abstract

Many technological solutions contain valuable components as waste and can become an additional source of rare-earth elements to meet the needs of modern production. The development of technologies based on commercially available and cheap sorbents reveals the possibility for rare earth recovery from various solutions. This paper provides research on using a combination of KU-2-8 and AV-17-8 ion exchangers in different molar ratios for cerium ions sorption from its nitrate solution. The mutual activation of the ion exchangers in an aqueous medium provides their transformation into a highly ionized state by the conformational and electrochemical changes in properties during their remote interaction. The ion exchange dynamics of solutions were studied by the methods of electrical conductivity, pH measurements, and atomic emission analysis of the solutions. The research showed that the maximum activation of polymers was revealed within the molar ratio of KU-2-8:AV-17-8 equal to 3:3. In more detail, in comparison to AV-17-8, this interpolymer system showed an increase in the sorption degree by more than 1.5 times after 6 h of interaction. Moreover, compared with KU-2-8, the same interpolymer system showed an increase in the degree of cerium ions sorption by seven times after 24 h of interaction. As a result, the total cerium ions sorption degree after 48 h of sorption by individual KU-2-8 and AV-17-8 was 38% and 44%, respectively, whereas the cerium ions sorption degree by the same interpolymer system in the molar ratio 3:3 became 51%. An increase in the sorption degree of cerium ions by the interpolymer system in comparison with individual ion exchangers can be explained by the achievement of a high ionization degree of ion exchangers being activated in the interpolymer system by the remote interaction effect.

## 1. Introduction

Rare-earth metals are used in various applications: in the chemical industry, in nuclear engineering, in metallurgy, etc. [1,2,3]. Cerium is a silvery-white rare-earth element that is easily forged and machined [4]. The main cerium deposits are located in the USA, Kazakhstan, Russia, Ukraine, Australia, and some other countries [5]. In metallurgy, cerium is used as an alloying addition. For example, its alloys with magnesium and aluminum increase strength at low density, and most often it is used in aircraft construction. The addition of only 1% of cerium to magnesium dramatically increases the latter’s tensile strength. Moreover, as an alloying addition to chromium and nickel alloys, cerium increases their heat resistance and durability [6]. Furthermore, cerium metal is a good “getter”, absorbing most gases (oxygen, hydrogen, nitrogen, carbon dioxide, etc.) and binding them through chemisorption. This metal is also widely used in pyrotechnic compositions, the powder of which is pyrophoric. In the chemical industry, cerium compounds are widely used as catalysts [7]. However, the availability of cerium on Earth is limited, and the demand for this element is growing annually [8]. Currently, many industrial enterprises have cerium content in their technological solutions as an associated product [9]. The recovery of cerium from technological solutions will provide an additional source of this valuable element.

Until now, the sorption and extraction methods for recovery of some metals have been successfully applied in hydrometallurgy [10]. Among the two methods, sorption methods are currently preferred due to a number of advantages over extraction. These methods are more environmentally friendly and have fewer technological cycles in comparison with the extraction technologies [11].

For the recovery of cerium from industrial solutions, adsorption processes using various materials, such as polymers, activated carbons, or biological materials, have recently sparked increased interest [12,13,14,15]. A common method for cerium ions sorption from solutions can be the use of commercially available and inexpensive ion exchangers: for example, KU-2-8 and AV-17-8 [16,17,18].

It is widely known that the KU-2-8 ion exchanger is mainly used for the isolation or separation of various metals [19], whereas the AV-17-8 ion exchanger basically is intended for waste and return water treatment [20]. For this reason, this paper investigates the use of KU-2-8 and AV-17-8 ion exchangers for cerium ions sorption from aqueous solutions. Based on our previous research [21] related to intergel systems, we decided to choose KU-2-8 and AV-17-8 ion exchangers for this study due to their availability and low cost. The combination of ion exchangers into an interpolymer system “KU-2-8–AV-17-8” with different molar ratios can be applied to investigate the remote interaction effect [22] on improving the process of cerium ions sorption. Due to the mutual activation of polymers during their remote interaction, they pass into a highly ionized state, which leads to a significant increase in the degree of metal ions sorption by the interpolymer system compared to the individual polymers. In this regard, the goal of this research was to study the ion exchange dynamics of cerium ions sorption by the interpolymer system “KU-2-8–AV-17-8” from cerium (III) nitrate solution.

## 2. Materials and Methods

### 2.1. Materials

For this research, the following materials were used: (1) strongly acidic KU-2-8 (H^+^ form) (Tokem, Russia), a crosslinked ion exchanger based on styrene and divinylbenzene (analog of Purolite C-100 (Lenntech, Delfgauw city, The Netherlands), Amberlite IR 120 (Lenntech, Delfgauw, The Netherlands), Dowex HGR-W2 (Lenntech, Delfgauw, The Netherlands), and Lewatit S-100 (Lenntech, Delfgauw, The Netherlands)); and (2) strongly basic AV-17-8 (OH^−^ form) (Tokem, Kemerovo, Russia), an anion exchanger based on a copolymer of styrene and divinylbenzene with benzyl trimethyl ammonium functional groups (analog of Dowex SBR C (Lenntech, Delfgauw, The Netherlands), Amberlite IRA-400 (OH) (Lenntech, Delfgauw, The Netherlands), and Duolite APA-366 (Lenntech, Delfgauw, The Netherlands)).

The following reagents were used: cerium (III) nitrate hexahydrate (99.999% trace metals basis, Sigma-Aldrich) as cerium ions source in solution, reagent arsenazo III (Sigma-Aldrich) in powder form as a color-forming reagent to determine cerium concentration, and perchloric acid (HClO_4_) (Sigma-Aldrich, Darmstadt, Germany) for standard solution preparation.

The following measurement instruments and equipment were used: conductometer MARK-603 (Vzor, Nizhny Novgorod, Russia) for the measurements of the specific electric conductivity of solutions, which is important for characterizing the equilibrium of polyelectrolytes dissociation and understanding the charge transfer by ions. The hydrogen ions concentration was determined by a Metrohm 827 pH-meter pH-Lab (Switzerland). Measurements of pH were provided to study the acid–base properties of the solution. The mass of the samples was measured using an analytical balance SHIMADZU AY220 (Shimadzu Corporation, Kyoto, Japan). The optical density measurements for the subsequent calculation of the cerium (III) concentration in solution was determined by a Jenway-6305 (Cole-Parmer, Jenway, York, UK) spectrophotometer. For the residual cerium ions detection from liquid samples, the Optima 8300DV inductively coupled plasma spectrometer (Perkin Elmer, Waltham, MA, USA) with a wavelength range 165–782 nm was used. Measurement errors did not exceed 1%.

### 2.2. Determination of the Polymers Swelling Coefficient

The ion exchangers KU-2-8 and AV-17-8 were put separately in the polypropylene meshes according to their molar ratios X:Y (6:0, 5:1, 4:2, 3:3, 2:4, 1:5, and 0:6), and each ion exchanger was placed in a glass with distilled water for 48 h for swelling. The swelling coefficient (*K_sw_*) was determined by the gravimetric method: the dry and swollen ion exchanger was weighed, and *K_sw_* was calculated by Equation (1).

The swelling coefficient was calculated by the following Equation (1):

(1) $$K_{sw}=\frac{m_{2}-m_{1}}{m_{1}},$$

where *m*_1_ and *m*_2_ (in g) are masses of the dry and swelled ion exchangers, respectively.

Upon reaching an equilibrium state in weight, the swollen ion exchangers were taken, and according to molar ratios, the interpolymer pairs were composed for further mutual activation.

### 2.3. Activation of the Interpolymer System

**Figure 1:**
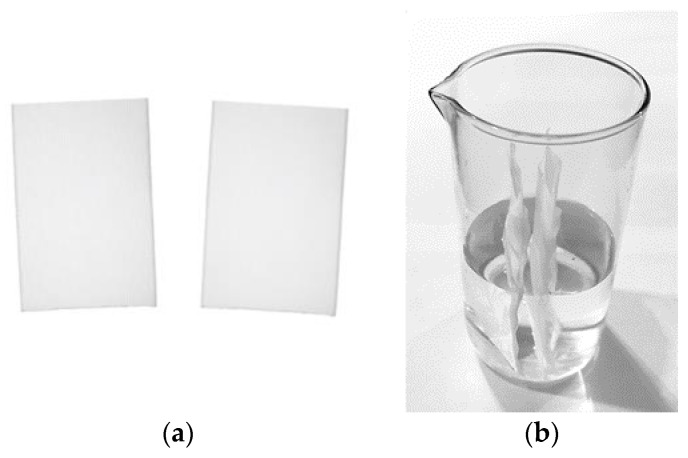
(**a**) Polypropylene meshes; (**b**) Illustration of the activation process of the interpolymer system.

The activation of the interpolymer system is required to transfer the ion exchangers into a highly ionized state by changing their conformational and electrochemical properties by remote interaction [14]. For activation, the polypropylene meshes with swollen ion exchangers inside (Figure 1a) were placed in a glass with distilled water at a distance of about 1–2 cm opposite each other, forming an interpolymer system “KU-2-8–AV-17-8” (Figure 1b).

**Scheme 1:**
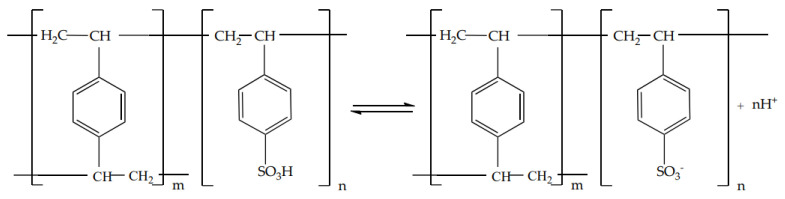
The chemical formula of KU-2-8 ion exchanger.

**Scheme 2:**
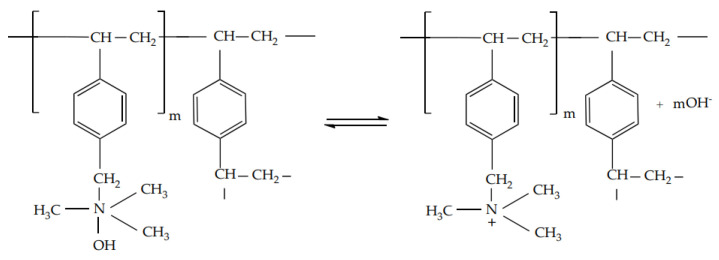
The chemical formula of AV-17-8 ion exchanger.

As known from [23], the acid–base properties of ion exchangers are associated with the dissociation of ion exchangers KU-2-8 (H^+^ form) and AV-17-8 (OH^−^ form) with the formation of free H^+^ and OH^−^ ions, respectively. As a result, free H^+^ (Scheme 1) and OH^−^ (Scheme 2) ions form water molecules leaving the functional groups of ion exchangers without counterions and being stabilized by intramolecular interactions. The electrochemical and conformational changes in macromolecules lead to an increased sorption capacity of the interpolymer system “KU-2-8–AV-17-8” in relation to cerium ions in comparison with individual ion exchangers.

Two main dissociation steps occurred in the aqueous solution.

1. Dissociation of acidic KU-2-8 ion exchanger (Scheme 1):

2. Dissociation of strongly basic AV-17-8 ion exchanger (Scheme 2):

### 2.4. Determination of the Polymer Chain Binding Degree

The polymer chain binding degree of internode links of the polymer chain was calculated according to Equation (2):

(2) $$\theta =\frac{\vartheta_{sorbed}}{\vartheta_{1}+\vartheta_{2}\;}\times 100\%,$$

where ϑ*_sorbed_* is the amount of sorbed cerium ions (in mol), $\vartheta_{1}$ is the amount of KU-2-8 (in mol), and $\vartheta_{2}$ is the amount of AV-17-8 (in mol). 

### 2.5. Plotting a Calibration Curve

The method for cerium ions determination was based on the formation of a colored complex compound of an organic analytical reagent arsenazo III with cerium ions. As known, cerium ions do not have a chromoform effect; therefore, to obtain an analytical form, it is necessary to use a colored reagent arsenazo III (bisazo-derivatives of chromotropic acid) [24].

To plot a calibration curve, a solution of cerium (III) nitrate hexahydrate with a concentration of 100 mg/L was prepared. After that, this solution was diluted to obtain the working solution with a concentration of 10 mg/L. Then, five standard solutions which contained 10, 20, 30, 40, and 50 µg of the analyte in 50 mL were prepared and transferred into the volumetric flasks with a capacity of 50 mL. Then, 12 mL of arsenazo (0.015%) and 2 mL of perchloric acid solution (0.08 M) were poured into each flask. Further, the volume of each solution was brought to 50 mL with distilled water, and after 15 min, the measurements were started. It should be noted that the reference solution contained all of the above components with the exception of the analyte.

**Figure 2:**
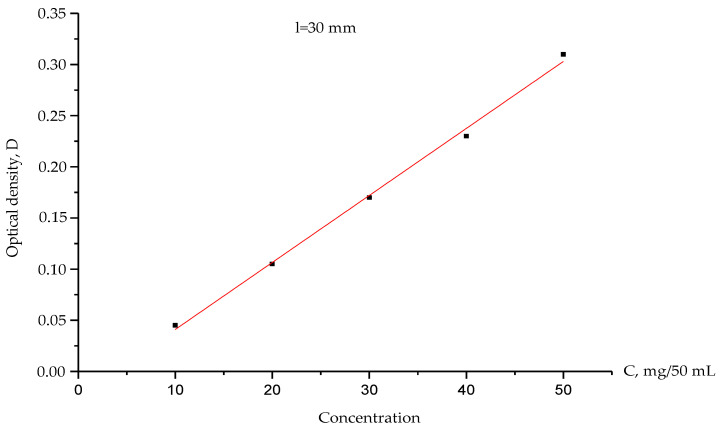
The calibration curve for determining the concentration of cerium (III) in the test solutions.

Therefore, to prepare a calibration curve (Figure 2), the five abovementioned standard solutions were prepared with a known cerium concentration. The analytical signal (optical density, D) was measured in each standard solution by the spectrophotometer. The measurement results were used to create a calibration curve in the coordinates of the analytical signal (optical density, D) and cerium concentration (C).

The optical density (D) of the formed cerium ions complex in the solutions was determined by the Jenway-6305 spectrophotometer. The calibration curve was plotted using the Origin software (r^2^ value is 0.99605, D = 0.0655C−0.0245).

### 2.6. Determination of Residual Concentration of Cerium Ions

For the experiments, 1000 mL of the cerium (III) nitrate hexahydrate solution (C = 100 mg/L) was prepared and poured into 7 glasses with 100 mL each. The ion exchangers were put separately into 2 polypropylene meshes (1 glass with solution) in accordance with their molar ratios X:Y (6:0, 5:1, 4:2, 3:3, 2:4, 1:5, and 0:6) to form the interpolymer system KU-2-8:AV-17-8 (X:Y). For spectrophotometer analysis, one aliquot (1 mL) was taken from each solution at the set time. The aliquot sampling times were 0.1, 0.5, 1, 2, 4, 6, 24, 40, and 48 h. Finally, 63 aliquots of solution were obtained.

For spectrophotometric analysis, each aliquot (1 mL) with an unknown concentration of the analyte was transferred into the volumetric flasks with a capacity of 50 mL. Then, 12 mL of arsenazo (0.015%) and 2 mL of perchloric acid solution (0.08 M) were poured into each flask. Furthermore, the volume of each solution was brought to 50 mL with distilled water, and after 15 min, the measurements were started. It should be noted that the reference solution contained all of the above components with the exception of the analyte.

The optical density measurements were provided for the analyzed solution with an unknown cerium ions concentration. Having obtained the value of the analytical signal (D) and using the prepared calibration curve (Figure 2), we found the unknown concentration corresponding to each signal. The optical density (D) of the solutions with cerium ions was determined by the Jenway-6305 spectrophotometer. Each measurement was repeated 3 times.

The sorption degree was calculated using the following Equation (3):

(3) $$\eta =\frac{C_{initial}-C_{residual}}{C_{initial}}\times 100\%,$$

where *C_initial_* and *C_residual_* are the initial and residual concentration (in g/L) of cerium ions in the solution, respectively.

## 3. Results and Discussion

### 3.1. Effect of the Swelling Degree and Molar Ratios of Ion Exchangers in the Interpolymer Systems on the Degree of Cerium Ions Sorption

The experiments showed that the swelling degree of the KU-2-8 (being in the interpolymer system) took the value *K_sw_* = 1.71; in comparison, this ion exchanger outside the interpolymer system had a swelling degree *K_sw_* = 1.31. Similarly, the swelling degree of the AV-17-8 took the value *K_sw_* = 1.70; in comparison, this ion exchanger outside the interpolymer system had a swelling degree *K_sw_* = 1.52. It should be emphasized that the swelling degree of polymers indicates an increase in their permeability due to the hydration of exchange groups, which leads to stretching of the polymer three-dimensional matrix and an increase in the cerium ions sorption [23]. Therefore, both the swelling degree of KU-2-8 and AV-17-8 ion exchangers and their remote interaction in the interpolymer system affected the changes in the conformational and electrochemical properties and led to the following increased sorption properties.

The polymer chain binding degree (Table 1) was calculated according to Equation (2), which indicates the number of units around the central metal ion and directly depends on the degree of ionization of the macromolecules during the remote interaction of ion exchangers. The maximum obtained value of the polymer chain binding degree (*θ*) was equal to 5.16% for the interpolymer system in the ratio of 3:3 after 48 h of interaction (Table 1). This means that out of 100 links of the polymer chain, about five binds to the cerium ions.

**Figure 3:**
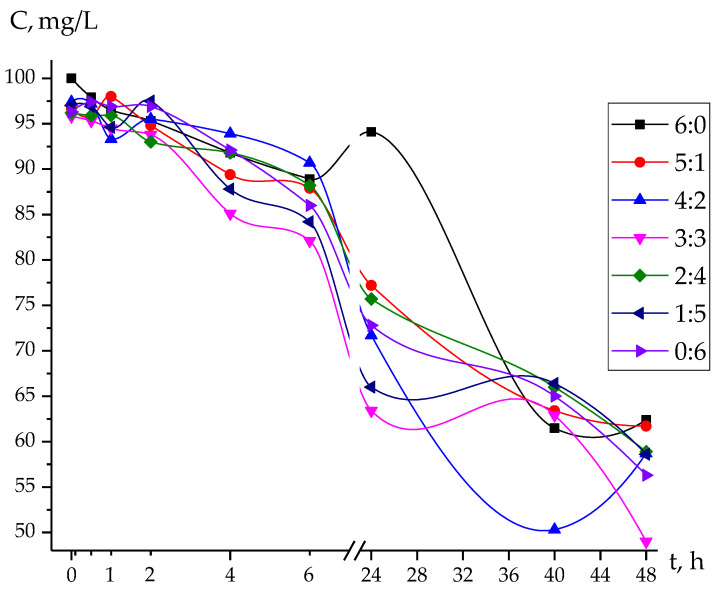
The residual concentration C of cerium ions in a solution after sorption as a function of time.

**Figure 4:**
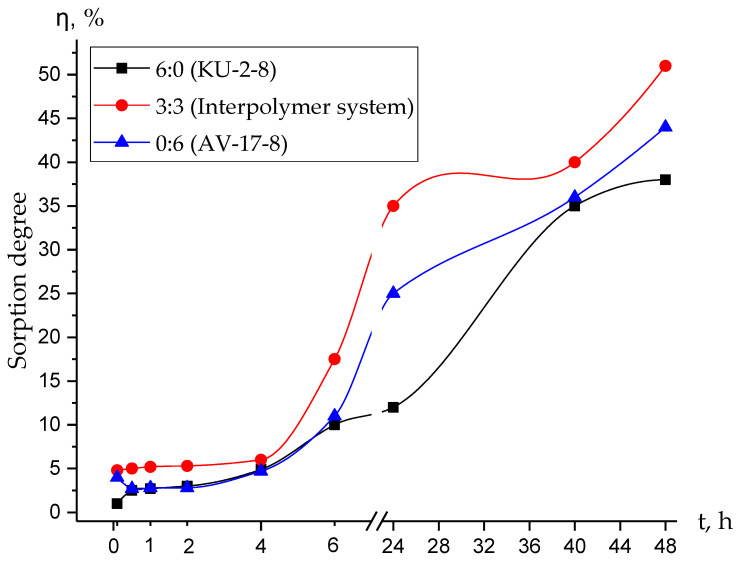
The dependence of the cerium ions sorption degree as a function of time.

According to experimental data on the residual concentration of cerium ions in solution (Figure 3), an interpolymer system KU-2-8:AV-17-8 (3:3) showed that the highest sorption activity and was selected for further comparison with individual KU-2-8 and AV-17-8 ion exchangers (Figure 4).

As seen from Figure 4, after 6 h of interaction, an increase in the sorption degree by more than 1.5 times of the interpolymer system KU-2-8:AV-17-8 (3:3) compared to the individual AV-17-8 was revealed, whereas after 24 h of remote interaction, the same interpolymer system showed an increase in the sorption degree relative to the individual KU-2-8 by seven times. According to the obtained results on the residual concentration, the highest sorption degree (51%) was observed after 48 h of the sorption process (Figure 3).

**Figure 5:**
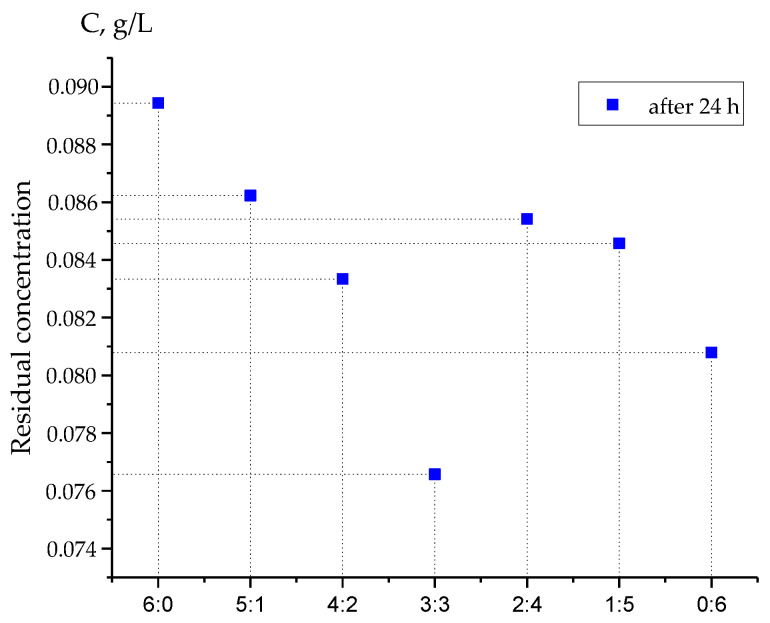
Atomic emission analysis of residual concentration of Ce ions in a solution after 24 h of sorption.

The activation of KU-2-8 and AV-17-8 ion exchangers showed that their swelling degree during the remote interaction was higher than the swelling degree outside the interpolymer system “KU-2-8–AV-17-8”. The increased swelling degree may indicate the improvement in the permeability of polymers being in the interpolymer system, which subsequently leads to an increase in the sorption degree due to the additional opening of the polymer coil. The data on the change in pH and electrical conductivity upon activation showed the influence of the remote interaction effect on both ion exchangers, which subsequently led to an increase in the cerium ions sorption by the interpolymer system “KU-2-8–AV-17-8” (3:3). The cerium ions residual concentration in the solution after the sorption process showed that the abovementioned interpolymer system showed the highest efficiency. The calculated data on the polymer chain binding degree (*θ*) in various ratios showed that the interpolymer system in the molar ratio of 3:3 has the highest value. The data on the sorption degree (η) showed the effectiveness of the interpolymer system in comparison with individual ion exchangers. The atomic emission analysis (Figure 5) confirmed the obtained data, where at a ratio of 3:3, low values of the residual concentration of cerium ions in the solution were observed. The obtained data confirm the increased sorption activity of the interpolymer system “KU-2-8–AV-17-8” (3:3) in relation to cerium (III) ions.

### 3.2. Electrochemical Investigations of KU-2-8:AV-17-8 Interpolymer System in Aqueous Solutions

**Figure 6:**
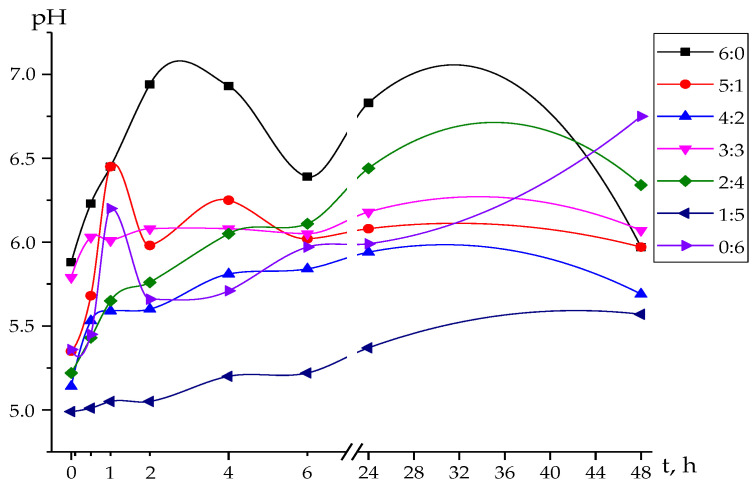
Changes in pH of a solution during the activation process as a function of time.

Figure 6 shows the changes of pH of aqueous solutions with interpolymer system KU-2-8:AV-17-8 in various molar ratios. An increase in the pH degree in each of the ratios could be explained by the binding of H^+^ protons and an increase in OH^−^. The interpolymer systems, in comparison with the initial KU-2-8 and AV-17-8 ion exchangers, had noticeable changes in pH values. At the initial stage, the pH of these interpolymer systems increased up to 4 h of interaction, which was due to the release of H^+^ ions into the solution as a result of the process of functional sulfo groups’ dissociation of the cation exchanger KU-2-8. After 24 h, the dissociation became weaker, except for the ratio of 0:6 (individual AV-17-8), where the additional dissociation of AV-17-8 (0:6) provided the release of OH^−^ anions into the solution till 48 h of activation process.

**Figure 7:**
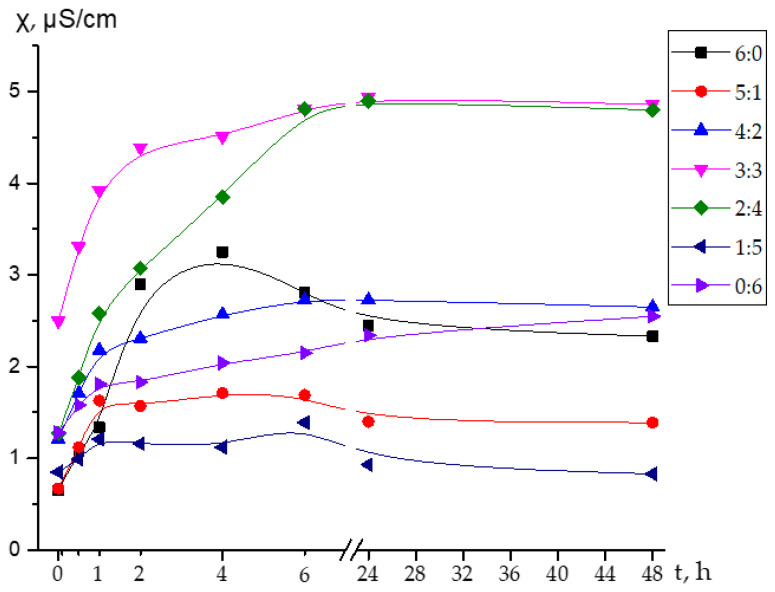
Changes in specific electrical conductivity of a solution as a function of time in the presence of ion exchangers during the activation process.

At the same time, considering the dependence of the change in the specific electrical conductivity of aqueous solutions on the molar ratio of KU-2-8:AV-17-8 in time (Figure 7), it could be noted that both in the interpolymer systems and the individual AV-17-8 (0:6), there was an increase in the values of specific electrical conductivity within the first 4 h of activation.

The increase in the specific electrical conductivity of aqueous solutions in the presence of interpolymer systems could be explained by the emergence of new OH^−^ ions due to the expansion of additionally dissociated AV-17-8 units. An increase in the specific electrical conductivity of an aqueous solution of the initial ion exchanger AV-17-8 could be due to the appearance of OH^−^ ions in the solution, as a result of the interaction of the polybase with water molecules. An excess of OH^−^ ions increases the specific electrical conductivity of the system.

It should be noted that in the interpolymer system KU-2-8:AV-17-8 (3:3), in comparison with other systems, a deeper mutual activation of polymers might occur through the transition of polymers to a highly ionized state, which leads to a significant increase in the sorption capacity of the interpolymer system KU-2-8:AV-17-8 (3:3).

According to Le Chatelier’s law, the additional dissociation of both polymers might occur. As a result of this process, due to the electrostatic repulsion of the -N^+^ groups in the AV-17-8 macromolecule, the links were unfolded and the intramolecular bonds were destroyed. When the intrachain links are destroyed, the macromolecule of AV-17-8 unfolds. The disclosed chains were additionally subjected to dissociation. Due to this, additional OH^−^ groups appear in the system, which causes an increase in the pH (Figure 7).

According to the specific electrical conductivity measurements, a slight increase in this parameter in time was revealed; the maximum values were reached after 48 h (Figure 7). Moreover, by analyzing the pH data of the solutions, it was supposed that the acidity of solutions increased most probably due to the replacement of H^+^ ions in the structure of KU-2-8 by cerium ions, thereby displacing the H^+^ ions into the solution. Comparing the swelling degree of polymers in distilled water and in a solution of cerium nitrate, it was supposed that in a cerium nitrate solution, the swelling degree became somewhat lower as sorption proceeded due to the fact that polymer units became less ionized and polymer molecules tended to a more energetically favorable form.

Figure 7 shows the change in specific electrical conductivity in the solutions during the activation process of the “KU-2-8–AV-17-8” interpolymer system at different molar ratios. In mostly all ratios, an increase in electrical conductivity values was observed, which might be explained by an increase in the OH^−^ medium in solutions, released by the additional dissociation of the strongly basic anion exchanger AV-17-8 [25]. A noticeable increase in the specific electrical conductivity values was observed for the interpolymer system “KU-2-8–AV-17-8” (3:3).

## 4. Conclusions

The obtained results demonstrate the potential of using interpolymer systems in rare-earth metal recovery. The remotely activated industrial ion exchangers showed an increase in sorption activity in comparison with the individual KU-2-8 (6:0) and AV-17-8 (0:6) ion exchangers. This research showed that the maximum activation of polymers was revealed within the molar ratio of KU-2-8:AV-17-8 equal to 3:3. An increase by more than 1.5 times in the sorption degree of this interpolymer system in comparison to the individual AV-17-8 (0:6) after 6 h of interaction was revealed, whereas after 24 h of remote interaction, the interpolymer system in a ratio of 3:3 showed an increase in the sorption degree relative to the individual KU-2-8 (6:0) by seven times. The total cerium ions sorption degree after 48 h of sorption by individual the polymers KU-2-8 and AV-17-8 was 38% and 44%, respectively, whereas the cerium ions sorption degree by the interpolymer system of KU-2-8:AV-17-8 (3:3) was 51%. An increase in the sorption degree of cerium ions by the interpolymer system “KU-2-8–AV-17-8” (3:3) in comparison with individual ion exchangers can be explained by the achievement of a high ionization degree of the interpolymer system activated by the remote interaction effect.

Ion exchange dynamics in the sorption process by interpolymer systems relative to the individual polymers is associated with some factors, one of which is conformational transformations that have a significant effect on the reaction mechanism. The highest sorption of cerium ions by the interpolymer system occurred at KU-2-8:AV-17-8 (3:3).

The obtained results indicate the possibility of the application of the interpolymer systems in highly efficient sorption technology for the sorption of rare-earth elements, including cerium, from industrial solutions, as well as in the processes of the concentration and separation of ions of various nature from aqueous systems for solving technological, environmental, and other problems. The obtained experimental results on the ion exchange dynamics might be used for the basis of the development of modern technologies for rare-earth metal ions recovery from industrial waste solutions.

## Figures and Tables

**Table 1:** The polymer chain binding degree at different ratios of ion exchangers (θ, %).

Time, h Ratio	0.1	0.5	1	2	4	6	24	40	48
6:0	0.11	0.22	0.37	0.48	0.88	1.19	0.63	4.16	4.06
5:1	0.35	0.45	0.21	0.56	1.79	1.30	2.46	3.95	4.19
4:2	0.29	0.47	0.75	0.51	0.69	1.05	3.20	4.63	4.68
3:3	0.46	0.31	0.61	0.69	1.62	0.19	4.08	4.14	5.16
2:4	0.42	0.45	0.45	0.78	0.91	1.31	2.71	3.79	4.58
1:5	0.03	0.35	0.62	0.27	1.38	1.79	3.84	3.75	4.69
0:6	0.49	0.29	0.35	0.35	0.89	1.58	3.08	3.96	4.95

## Data Availability

Data Sharing is not applicable.
